# Swelling and Degrafting of Poly(3-sulfopropyl methacrylate) Brushes

**DOI:** 10.1021/acs.langmuir.4c02714

**Published:** 2024-09-30

**Authors:** Sabrina Sant, Kuljeet Kaur, Harm-Anton Klok

**Affiliations:** † Institut des Matériaux and Institut des Sciences et Ingénierie Chimiques, Laboratoire des Polymères, École Polytechnique Fédérale de Lausanne (EPFL), Station 12, CH-1015 Lausanne, Switzerland; ‡ National Center of Competence in Research Bio-inspired Materials, Chemin des Verdiers 4, CH-1700 Fribourg, Switzerland

## Abstract

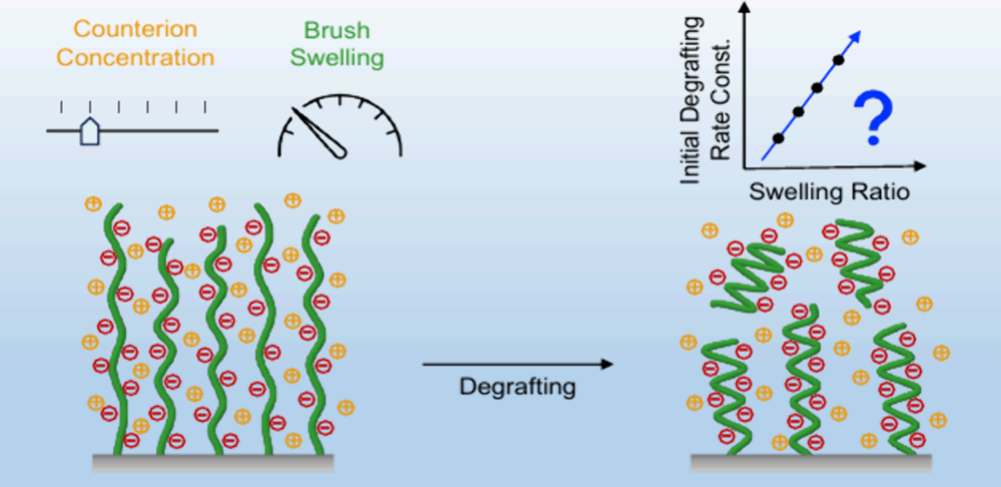

Upon exposure to a good solvent, polymer brushes prepared via surface-initiated polymerization can undergo degrafting via cleavage of bonds that anchor the polymer tethers to the underlying substrate. As polymer brushes are often used in a solvent swollen state, this has implications for the longevity of these polymer coatings. Improving the fundamental understanding of this process is thus also of practical importance. It is believed that degrafting is the consequence of tension amplification at the bonds that anchor the polymer grafts, which is driven by swelling of the polymer brush film. Taking advantage of the sensitivity of the swelling behavior of poly(3-sulfopropyl methacrylate) (PSPMA) brushes toward changes in ionic strength, this study has investigated the degrafting behavior of these brushes in aqueous media at different LiCl and NaCl concentrations. The aim of these experiments was to investigate whether the rate constant of the degrafting process was correlated with the swelling ratio of the PSPMA brushes. The experiments show that in aqueous LiCl solutions, the initial rate constant of the degrafting process is correlated with the swelling ratio of the PSPMA brush. This observation represents a first example of the correlation between these two parameters for hydrophilic polymer brushes in aqueous media and supports the idea that degrafting is a mechanochemical process driven by a swelling-induced tension at the polymer–substrate interface.

## Introduction

**Figure 1:**
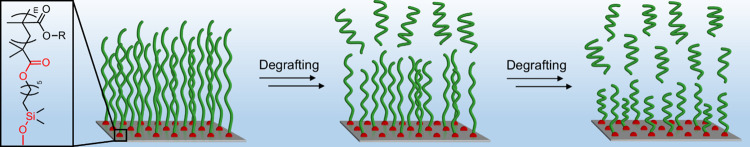
Schematic illustration of the degrafting of polymer brushes upon immersion in a good solvent or exposure to solvent vapor.

Surface-initiated polymerization reactions allow the preparation of densely grafted, chain-end-tethered polymer thin films, which are also referred to as polymer brushes.^1−4^ Degrafting of polymer brushes refers to the successive detachment of chain-end anchored polymer tethers as a consequence of cleavage of bonds that attach the polymer grafts to the underlying substrate (Figure 1).^5^ Generally, and in this article, degrafting is considered as a spontaneous process that occurs when a polymer brush film is placed in contact with a good solvent. As illustrated in Figure 1, degrafting results in a gradual decrease in grafting density, and thus can be monitored by following the dry film thickness of a polymer brush as a function of time. This phenomenon was first reported in 2008 for poly(poly(ethylene glycol) methacrylate) (PPEGMA) brushes upon incubation in cell culture medium,^6^ but since then has been observed and reported for a broad range of other hydrophilic polymer brushes.^7−10^ In addition to liquid media, degrafting has also been observed upon exposure of hydrophilic polymer brushes to humid air.^11^ The effect of chain extension on bond tension amplification of surface-attached polymers has also been subject of theoretical and simulation studies.^12−14^ The general hypothesis is that degrafting is the consequence of a tension amplification at the bonds that anchor the polymer grafts, which is driven by swelling of the polymer brush film.^5^ In aqueous or water-containing media, the swelling-driven tension amplification is believed to, in a mechanochemical sense, lower the energy barrier required for the hydrolysis of siloxane, ester or amide bonds that are typically incorporated in the anchoring motifs that are used to grow polymer brushes via surface-initiated polymerization. Note that in this article, and also often more generally in the literature, degrafting is used to describe the spontaneous process in which water molecules act as the reagent, as opposed to the cleavage of brushes with more reactive reagents, such as e.g. HF or TBAF,^2,4^ that are frequently used for the quantitative cleavage of polymer brushes for molecular weight analysis. Degrafting is not only a fundamental problem, but has also technological relevance, since polymer brushes are often used in a solvated state, and degrafting thus is related to the robustness and longevity of polymer brush coatings.

As mentioned above, degrafting is believed to be driven by a tension that is the consequence of swelling of the polymer brush film. Swelling of polymer brushes is often described in terms of the swelling ratio (α), which is defined as the film thickness of a brush in the solvated state (*h*_swollen_), divided by the dry film thickness (*h*_dry_). First experimental evidence that highlighted the correlation between swelling of a polymer brush and the degrafting process was obtained in a study that investigated hydrophobic poly(*tert-*butyl methacrylate) (P*t*BMA) brushes in organic media.^15^ By using water-miscible, organic solvents to which a small quantity of water was added, it was possible to tune the swelling ratio of the P*t*BMA brushes, and to systematically investigate the degrafting of these films as a function of swelling ratio. These experiments revealed that the rate constant that describes the degrafting reaction was correlated with the swelling ratio of the polymer brushes, which supports the hypothesis that degrafting is a mechanochemical process driven by a swelling-induced tension at the polymer–substrate interface.

While the experiments with the hydrophobic P*t*BMA brushes discussed above provided first evidence of the impact of swelling on the degrafting of polymer brushes, most of the reported observations of degrafting involve hydrophilic polymer brushes in aqueous media. A challenge with studying the influence of swelling on degrafting is to design experiments that allow to systematically vary swelling of a polymer brush by changing the solvent conditions. For hydrophobic polymer brushes, this can be accomplished by changing the nature of the organic solvent. To investigate the impact of swelling on the degrafting of hydrophilic brushes in aqueous media, other strategies are needed. An interesting possibility is to explore the effect of ionic strength on the swelling properties of polyelectrolyte brushes.^16−18^ The influence of ionic strength on the swelling behavior of both weak polyelectrolyte brushes, such as for example poly(acrylic acid) (PAA)^19,20^ and poly(2-(diethylamino)ethyl methacrylate) (PDEA)^21^ brushes, as well as strong polyelectrolyte brushes such as poly(styrene sulfonate) (PSS),^22^ poly([2-(methacryloyloxy)ethyl]trimethylammonium chloride) (PMETAC),^22,23^ and poly(3-sulfopropyl methacrylate) (PSPMA)^22^ brushes, is well documented. This potentially provides opportunities to study the degrafting of hydrophilic polymer brushes in aqueous media across a range of swelling ratios that are controlled by modulation of the ion strength.

In a first attempt to study the degrafting of hydrophilic polymer brushes in aqueous media as a function of swelling ratio, this study has investigated the swelling behavior of PSPMA brushes in aqueous LiCl and NaCl solutions that cover a range of salt concentrations. Degrafting experiments performed on PSPMA brushes in LiCl solutions revealed a positive correlation between the rate constant of the degrafting reaction and the swelling ratio of the PSPMA brushes, which represents a first example of the correlation between these two parameters for hydrophilic polymer brushes in aqueous solution, and also provides further support for the idea that degrafting is a mechanochemical process driven by a swelling-induced tension at the polymer–substrate interface.

## Experimental Section

### Materials

All chemicals were used as received unless stated otherwise. l-Ascorbic acid (99%), α-bromoisobutyryl bromide (BiBB, 98%), copper(II)chloride (99.999%), magnesium sulfate (anhydrous, ≥ 97%), *N*,*N*,*N*′,*N*″,*N*″-pentamethyl-diethylenetriamine (PMDETA), and 3-sulfopropyl methacrylate potassium salt (SPMA) (98%) were purchased from Sigma-Aldrich. Dimethylchlorosilane (DMCS, 99.5%) was purchased from abcr. 5-Hexen-1-ol (99%) and triethylamine (99%) were purchased from Acros Organics. Platinum on carbon (Pt/C, 10 wt % Pt, dry powder) was purchased from Fluorochem. Triethylamine was dried over KOH pellets before use. Dichloromethane (DCM) and toluene were purified and dried using a PureSolv solvent-purification system. Ultrapure water was obtained from a Millipore Direct-Q5 or a Milli-Q IQ 7003 water purification system. Silicon wafers (orientation <100>) were obtained from the Center of MicroNanoTechnology (CMi) at EPFL. The wafers were diced into pieces of 70 × 25 mm^2^.

### Methods

#### Nuclear Magnetic Resonance (NMR) Spectroscopy

^1^H and ^13^C NMR spectra were recorded as described in an earlier report.^24^

#### Ellipsometry

A variable angle spectroscopic ellipsometer from Semilab (SemiLAB SE2000) was used to measure dry and swollen film thicknesses. The experiments were performed at ambient conditions (temperature = ∼23 °C and humidity = ∼60%). The data was recorded at an incident angle of 70° and between wavelengths of 245–990 nm. The Spectroscopic Ellipsometry Analyzer (SEA) v1.6.1 software from Semilab was used to fit the ellipsometric data. Dry film thicknesses were analyzed using a four-layer model composed of silicon/silicon oxide/polymer brush/air. The complex refractive index spectra used for the silicon and silicon oxide layers were supplied with the SEA v1.6.1 software. The refractive index (*n*) of the polymer layer is described by the Cauchy approximation (*n* = *A* + *B*/λ2). To determine the layer thickness, the refractive index at an infinitely large wavelength *n*_inf_ (Cauchy A parameter) was kept constant at 1.46,^25^ while allowing the software to fit the Cauchy B parameter and the polymer layer thickness. The raw ellipsometric data of a dry PSPMA film with the corresponding fits are shown in Supporting Information Figure S1. The 70 × 25 mm^2^ PSPMA brush modified substrates were fully mapped with 81 points. For the degrafting experiments, 3 points were measured per substrate. The ellipsometric dry film thicknesses are reported as the average ± standard deviation (SD).

Film thicknesses of solvent-swollen polymer brushes were measured in a liquid cell at an incident angle of 70°. The samples were allowed to equilibrate in the liquid cell for 30–60 s before measuring one spot per substrate. The swollen thickness was measured on three substrates, and the reported values represent the average ± standard deviation (SD) of the three substrates. The swollen film thicknesses were determined with a four-layer model composed of silicon/silicon oxide/polymer brush/solvent. Complex refractive index spectra supplied by the SEA v1.6.1 software were used for the silicon, silicon oxide, and solvent (H_2_O) layers. The polymer brush layer was modeled by employing a Cauchy layer and *a* “gradient phase” layer in the SEA v1.6.1 software. Two sublayers were used in the gradient phase. A parabolic function (*c*(*z*) = *a*(*z* – *z*_0_)2 + *c*_0_, where *c* is the concentration of the top layer, and *z* the phase thickness) was used to model the transition concentration profile from the top layer to the bottom layer. The parabola parameters of *a* = −1.1999, *c*_0_ = 0.8598, *z*_0_ = 0.0939 were used to model all swollen film thicknesses. To model the polymer phase thickness, the Cauchy layer thickness was set to 0 nm with a refractive index *n*_inf_ of 1.46, while fitting the thicknesses and complex refractive indices of the two sublayers in the gradient phase. The complex refractive indices of the sublayers result through an effective medium approximation (EMA) of the adjacent layers above and below. A visualization of the gradient phase and the parabolic function to describe the polymer layer can be found in Supporting Information Figure S2, and raw ellipsometric data of a swollen PSPMA film with the corresponding fits are shown in Supporting Information Figure S3.

Swelling ratios (α) were calculated according to eq 1 and are reported with an error calculated using error propagation with the standard deviations of the dry and swollen film thicknesses.
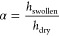
1

#### Determination of pH

The pH of aqueous solutions was determined with a Mettler Toledo SevenEasy potentiometric pH meter.

### Procedures

#### 5-Hexen-1-yl-2-bromo-2-methylpropionate

This compound was synthesized following a published procedure.^24^^1^H NMR (400 MHz, CDCl_3_-*d*) δ = 5.80 (ddt, *J* = 16.9, 10.2, 6.6 Hz, 1H, C*H*=CH_2_), 5.07–4.93 (m, 2H, CH=C*H*_2_), 4.18 (t, *J* = 6.5 Hz, 2H, OC*H*_2_), 2.15–2.04 (m, 2H, C*H*_2_CH=CH_2_), 1.93 (s, 6H, 2 C*H*_3_), 1.70 (m, 2H, C*H*_2_CH_2_O), 1.55–1.44 (m, 2H, C*H*_2_CH_2_CH) (Supporting Information Figure S4).

#### 6-(Chlorodimethylsilyl)hexyl 2-bromo-2-methylpropanoate

This compound was synthesized following a published procedure.^24^^1^H NMR (400 MHz, CDCl_3_-*d*) δ = 4.17 (t, *J* = 6.6 Hz, 2H, OC*H*_2_), 1.93 (s, 6H, 2 C*H*_3_), 1.67 (m, 2H, C*H*_2_CH_2_O), 1.54–1.23 (m, 6H, 3 C*H*_2_), 0.88–0.75 (m, 2H, C*H*_2_Si), 0.40 (s, 6H, Si(C*H*_3_)_2_) (Supporting Information Figure S5). ^13^C NMR (101 MHz, CDCl_3_-*d*) δ = 171.88 (*C*=O), 66.21 (O*C*H_2_), 56.13 (C(O)*C*), 32.59 (*C*H_2_CH_2_CH_2_Si), 30.93 (*C*H_3_), 28.36 (*C*H_2_CH_2_O), 25.56 (*C*H_2_CH_2_CH_2_O), 23.00 (*C*H_2_CH_2_Si), 19.02 (*C*H_2_Si), 1.81 (Si(*C*H_3_)_2_ (Supporting Information Figure S6).

#### Initiator Immobilization

Silicon wafers (70 × 25 mm^2^) were cleaned by sonication in ethanol, water, and acetone (5 min each), and subsequently dried under a flow of nitrogen. Prior to immobilization of the initiator, the wafers were exposed to O_2_ plasma for 30 min using a Femto plasma cleaner (Diener Electronic) at 200 W. After that, the wafers were placed in a holder, which was immersed in a solution of 6-(chlorodimethylsilyl)hexyl 2-bromo-2-methylpropanoate (10–15 mM) in dry toluene (90 mL). The solution was stirred at 1000 rpm under nitrogen at room temperature overnight. The modified wafers were washed with toluene, methanol and dried under a flow of nitrogen. The wafers were stored under vacuum until their use.

#### SI-ARGET ATRP of SPMA

A glass bottle containing an initiator modified 70 × 25 mm^2^ wafer was purged for at least 30 min with nitrogen while the monomer and catalyst solutions were prepared. The monomer solution was prepared in a Schlenk flask by dissolving SPMA (11.1 g, 4.5 mmol) and ascorbic acid (125 mg, 0.71 mmol) in water (8.3 mL) under sonication. Then, MeOH (15.3 mL) was added to the above mixture to yield the monomer solution. In a separate Schlenk flask, a catalyst stock solution of CuCl_2_ (33 mg, 0.024 mmol) and PMDETA (100 μL, 0.047 mmol) in MeOH (10 mL) was prepared. Three freeze–pump–thaw cycles were performed on the catalyst solution, and two on the monomer solution. After that, 1.4 mL of the CuCl_2_/PMDETA solution were added to the monomer solution, mixed, and then cannula transferred to the glass bottle with the wafer. The polymerization was allowed to proceed at room temperature while shaking on a plate shaker at 80 rpm for 2 h. The reaction was quenched by adding methanol, and the wafers were subsequently removed from the reaction medium, and washed extensively with DMSO, DCM and acetone, then dried with a stream of nitrogen and stored under vacuum until further use.

#### Degrafting Studies

One large (25 × 70 mm^2^) PSPMA brush-modified silica wafer was used for each experiment. The wafer was cut into smaller pieces of approximately 8 × 10 mm^2^, which were subsequently incubated in 3 mL of NaCl and LiCl solutions in MilliQ water with salt concentrations ranging from 5 to 500 mM at 37 °C for a defined amount of time. For each condition, 3 wafer pieces were used and followed throughout the experiment. After incubation, the wafers were removed and washed with water and methanol, then dried with a stream of nitrogen. Then, the ellipsometric dry film thickness was determined on three points per wafer. This process was repeated, by placing the wafers back in the appropriate solution and allowed to incubate further for a defined amount of time. The ellipsometric dry film thickness was measured after 0.5, 1, 1.5, 2, 3, and 5 h.

#### Statistical Analyses

The data are presented as mean ± standard deviation of the mean. Horizontal lines highlight significant differences. Stars (*, ** or ***) represent the results of Student’s *t*-test statistical analyses. Differences between the data are considered statistically significant at *p < 0.05, **p < 0.01, and ***p < 0.001.

## Results and Discussion

### Preparation of PSPMA Brush Modified Silicon Wafers

**Scheme 1:**
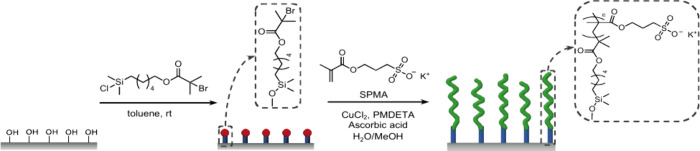
Preparation of PSPMA Brushes via SI-ARGET ATRP

The preparation of PSPMA brush-modified silicon wafers is presented in Scheme 1. For the experiments in this paper, PSPMA brushes were grafted from two 25 × 70 mm^2^ sized silicon wafers. The silicon wafers were first treated with oxygen plasma, and then modified with the initiator by immersion in a 10–15 mM solution of 6-(chlorodimethylsilyl)hexyl 2-bromo-2-methylpropanoate in dry toluene overnight. Subsequently, PSPMA brushes were grown using an ARGET ATRP protocol using CuCl_2_/PMDETA and ascorbic acid in a water/methanol mixture for 2 h. To assess the dry film thickness and uniformity of the PSPMA brushes, ellipsometry was used to map the entire surface area of the coated silicon wafers. Supporting Information Figure S7 presents the contour plots obtained from ellipsometry of the two 25 × 70 mm^2^ sized PSPMA brush-covered wafers. The contour maps confirm that SI-ARGET ATRP of SPMA results in uniform polymer brush coatings that cover the entire silicon wafer substrates. From these ellipsometric analyses, the dry film thicknesses of the PSPMA brushes were determined as 66.9 ± 1.5 nm (**wafer 1**) and 56.4 ± 1.4 nm (**wafer 2**).

### Swelling Properties

**Figure 2:**
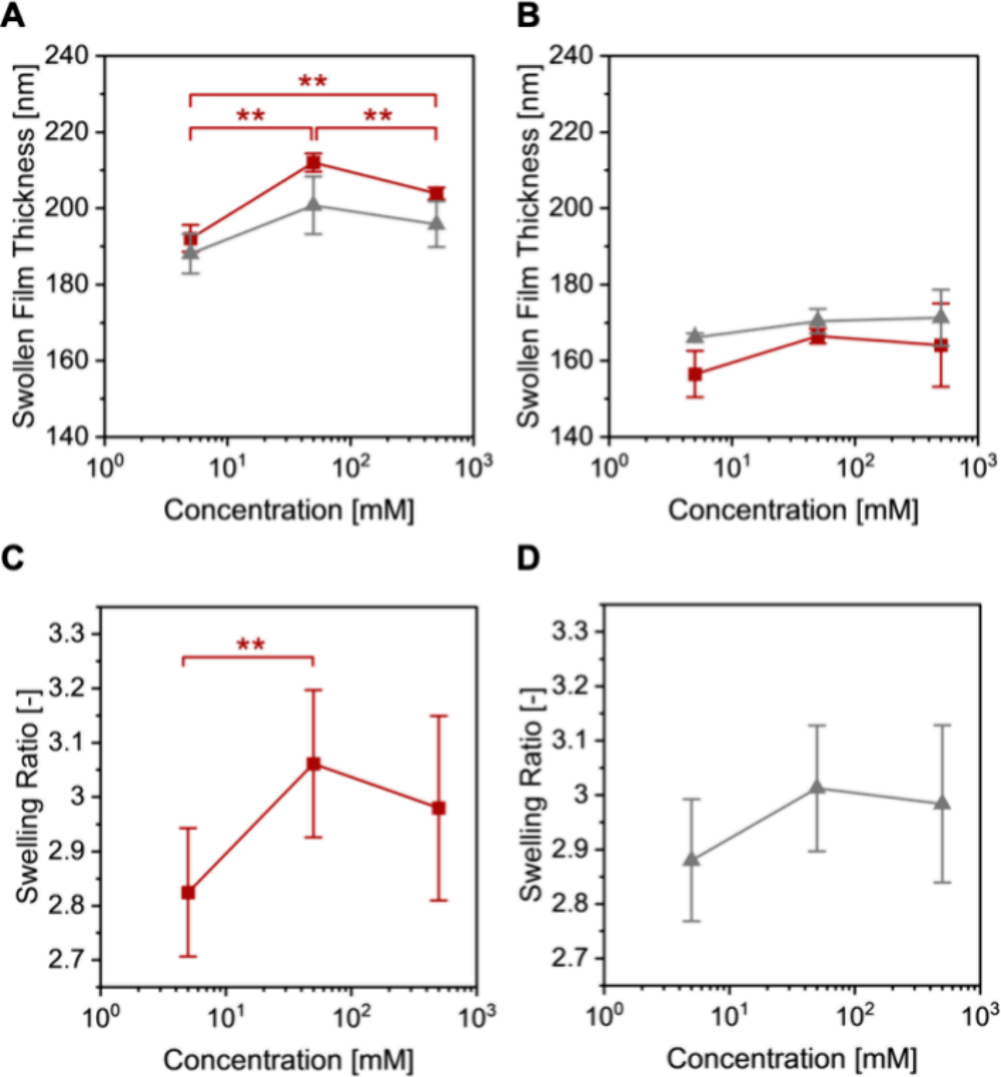
Swollen film thicknesses of PSPMA brushes originating from **wafer 1** (A) and **wafer 2** (B) measured in aqueous LiCl (squares) and NaCl (triangles) solutions with salt concentrations of 5, 50, and 500 mM. Average swelling ratio of PSPMA brushes derived from **wafer 1** and **wafer 2** in aqueous LiCl (C) and NaCl (D) solutions with salt concentrations of 5, 50, and 500 mM.

To study the swelling behavior of the PSPMA brushes, and probe the influence of ionic strength, 9 pieces of ∼8 × 10 mm^2^ sized substrates, which were obtained from the PSPMA brush coated 25 × 70 mm^2^**wafer 1** (*h*_dry_ = 66.9 ± 1.5 nm) and **wafer 2** (*h*_dry_ = 56.4 ± 1.4 nm) were used. The swelling properties of the PSPMA brushes were studied in aqueous solutions containing LiCl and NaCl with salt concentrations of 5, 50, and 500 mM. To determine the swollen film thicknesses, the substrates were placed in the corresponding salt solution, and allowed to equilibrate for approximately 30 s. Then, the swollen film thickness was determined by ellipsometry via a measurement on a single point per substrate. To obtain the swollen film thicknesses from the ellipsometry data, the Cauchy model was used by applying a parabolic gradient model to the polymer brush layer. For each salt, and for each salt concentration, the swollen film thickness of PSPMA brushes grafted from **wafer 1** and **wafer 2** was determined on 3 independent 8 × 10 mm^2^ samples from each wafer. Figure 2A,B plots the swollen film thicknesses of PSPMA brushes grown from **wafer 1** and **wafer 2** as a function of the LiCl and NaCl concentration. The observed effects of salt concentration on the swollen film thickness of the brushes are characteristic for PSPMA (and other strong polyelectrolyte brushes), and reflect increased swelling in the presence of moderate salt concentrations, followed by a collapse at very high salt concentration.^22^ For PSPMA brush-modified **wafer 1** the swollen film thickness increased from 192 ± 4 to 212 ± 2 nm upon increasing the LiCl concentration from 5 to 50 mM LiCl, and then decreased to 204 ± 2 nm upon further increasing the salt concentration to 500 mM. For PSPMA brushes grafted from **wafer 2** the film thickness changed from 157 ± 6 to 168 ± 2 and finally to 164 ± 11 nm when the LiCl concentration was varied from 5 to 50 and 500 mM. In NaCl solution, swollen film thicknesses of 188 ± 5, 201 ± 8, and 196 ± 6 nm were determined for PSPMA brushes grafted from **wafer 1** when the salt concentration was changed from 5 to 50, and to 500 mM. For PSPMA brushes that were grown from **wafer 2**, swollen film thicknesses of 166 ± 1, 170 ± 3, and 171 ± 7 nm were obtained at NaCl concentrations of 5, 50, and 500 mM. As indicated by the results of the statistical analysis in Figure 2A, PSPMA brushes grafted from **wafer 1** undergo statistically significant changes in swollen film thickness when the LiCl concentration is changed from 5, to 50 to 500 mM. For the other data points, the standard deviations are large but the results reveal similar trends with respect to the observed changes in swollen film thickness as a dependence of salt concentration. While these results underline the sensitivity of the swollen film thickness of polyelectrolyte brushes toward variations in salt concentration, they also highlight the challenges and uncertainties related to the determination of swollen film thicknesses of polymer brushes using ellipsometry.

From the swollen film thicknesses (*h*_swollen_), the swelling ratio, which is defined as the ratio of the swollen and dry film thickness, was determined from the average dry film thickness obtained by contour mapping of the entire synthesized wafer (either **wafer 1** or **wafer 2**), and the swollen film thickness (which for each salt and salt concentration is presented as the average of three, smaller 8 × 10 mm^2^ wafer substrates). Figure 2C,D shows the swelling ratio of the PSPMA brushes, represented as the average of **wafer 1** and **wafer 2**, as a function of LiCl and NaCl concentration, respectively. The swelling ratio of PSPMA brushes in LiCl varied from 2.82 ± 0.12 to 3.06 ± 0.09, and in NaCl from 2.88 ± 0.11 to 3.01 ± 0.12. As highlighted by the results of the statistical analysis, PSPMA brushes undergo a statistically significant increase in swelling ratio when the LiCl concentration in the aqueous medium is increased from 5 to 50 mM (Figure 2C). While the standard deviations of the other data points are large, as the data represent the average of two independent wafers, the results in Figure 2C,D indicate that the swelling ratio of the PSPMA brushes can be tuned, and follows the same trend as a function of salt concentration as the swollen film thicknesses illustrated in Figure 2A,B.

### Degrafting Experiments

The degrafting behavior of the PSPMA brushes was studied with the same nine ∼8 × 10 mm^2^ sized samples derived from **wafer 1** and **wafer 2** that were also used to determine the swollen thicknesses. For the degrafting experiments, a substrate was placed in a vial, and approximately 3 mL of an aqueous solution containing 5, 50, or 500 mM LiCl or NaCl, which had been preheated to 37 °C, was added. The vial was then capped and placed in an oven at 37 °C. After a specific period of time, the substrates were removed from the aqueous media, washed with water and methanol, and dried with a stream of nitrogen. Subsequently, the dry film thickness of the PSPMA brush was measured by ellipsometry. Finally, the substrates were placed back into the same degrafting media, which was maintained at 37 °C, and incubated again for a defined amount of time. The pH of the used salt solutions was checked, and found to be stable between 6.5–7 within the time frame of the experiment.

**Figure 3:**
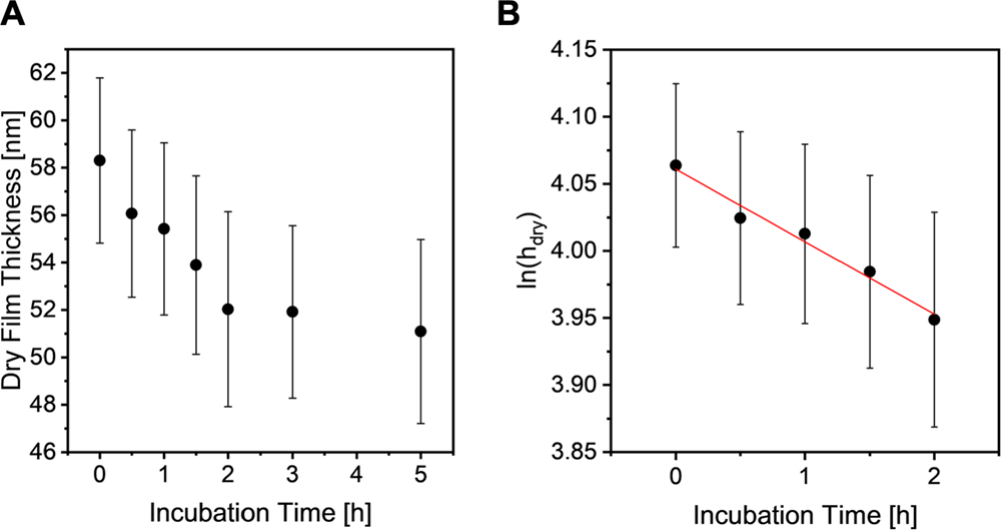
(A) Evolution of dry film thickness as a function of time for a PSPMA brush (**wafer 1**) incubated in a 5 mM aqueous NaCl solution. (B) Natural logarithm of the dry film thickness (*h*_dry_) of a PSPMA incubated in 5 mM aqueous NaCl as a function of time. The red line represents the result of linear regression analysis of the first 5 data points that are used to determine the initial rate constant of the degrafting reaction.

To determine the initial rate constant of the degrafting reaction, the evolution of the dry film thickness of the PSPMA brushes was followed as a function of the incubation time. As a typical example, this is illustrated in Figure 3A for PSPMA brushes derived from **wafer 1** incubated in a 5 mM NaCl solution. The results presented in Figure 3A also show that degrafting, as it is well-known from other work, does not result in complete cleavage of all surface-anchored polymer tethers, but instead that the rate of degrafting after an initial rapid decrease in dry film thickness slows down to reach a plateau dry film thickness.^5−11^ Considering that degrafting can be described as a pseudo-first-order kinetic process,^15^ the initial rate constant of the degrafting reaction (*k*_init_) can be obtained as the slope of the initial data points of a graph that plots ln(*h*_dry_) as a function of the incubation time (Figure 3B). For the data presented in Figure 3A this analysis affords an initial degrafting rate constant of 0.0054 ± 0.005 h^–1^. Supporting Information Figure S8, Supporting Information Figure S9 and Supporting Information Table S1 present the evolution of dry film thickness, respectively, ln(*h*_dry_) as a function of incubation time as well as the corresponding rate constants for all investigated PSPMA samples in aqueous LiCl and NaCl solutions with salt concentrations of 5, 50, and 500 mM.

**Figure 4:**
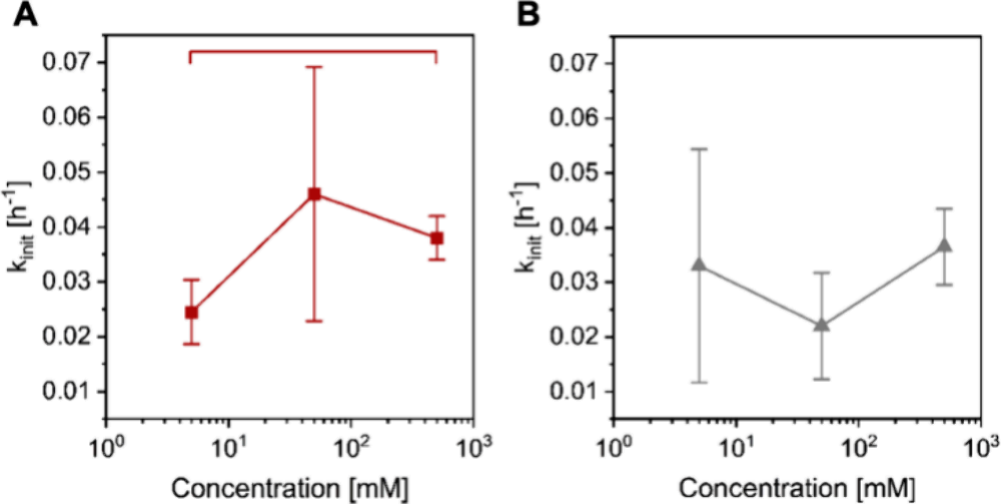
Average initial rate constants of degrafting for PSPMA brushes determined in aqueous LiCl (A) and NaCl (B) solutions with concentrations of 5, 50, and 500 mM. The error bars correspond to the error of the linear regression.

The average initial rate constants for degrafting of PSPMA brushes at different LiCl and NaCl concentrations are shown in Figure 4. Initial rate constants for the degrafting of PSPMA brushes in aqueous LiCl solutions range from 0.025 ± 0.006 to 0.046 ± 0.023 h^–1^, whereas in NaCl solutions rate constants vary from 0.022 ± 0.010 to 0.037 ± 0.007 h^–1^. While the standard deviations of the rate constants determined in aqueous NaCl solution do not allow to draw statistically relevant conclusions, the results of the experiments in LiCl solution presented in Figure 4A indicate a significant difference between the rate constant at 5 and 500 mM salt, and suggest a positive correlation between the rate constant of the degrafting reaction and the salt concentration. The results presented in Figure 4, which are related to the data and data analysis in Figure 3, also highlight the challenges to quantitatively study the degrafting of polymer brushes, and underline the importance of analyzing multiple samples.

**Figure 5:**
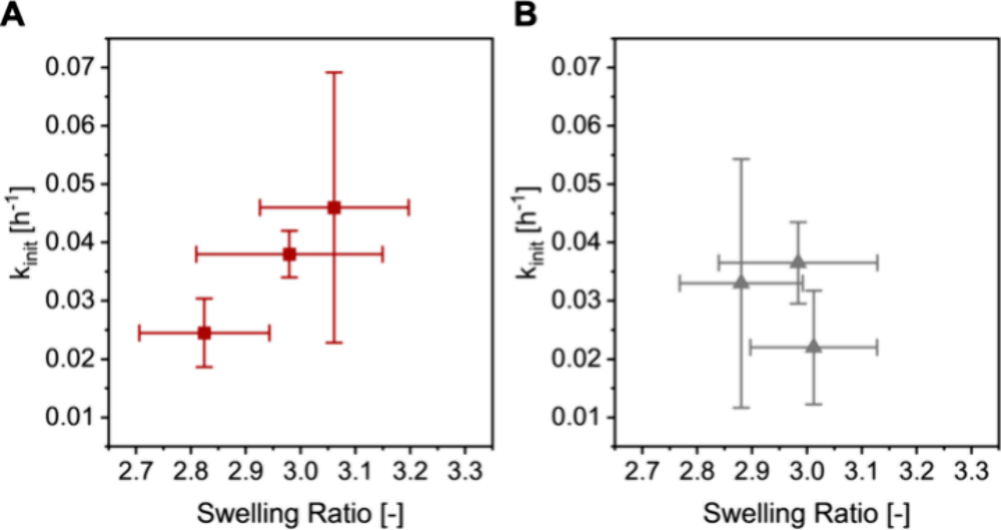
Initial degrafting rate constant (*k*_init_) for the degrafting of PSPMA brushes in LiCl (A) and NaCl (B) solution as a function of the swelling ratio of the brushes in the respective media.

Figure 5, finally, presents the initial rate constants for the degrafting of the PSPMA brushes at the 3 different investigated salt concentrations versus the initial swelling ratio, which is a measure for chain stretching and tension at the bonds at the brush–substrate interface,^5,15^ in the same medium. While the standard deviations in the initial rate constants and swelling ratios that are determined in NaCl solutions are too large to draw any significant conclusions (Figure 5B), the data in Figure 5A that are obtained by comparing degrafting and swelling of PSPMA brushes in aqueous LiCl solutions point toward a positive correlation between the rate constant for the degrafting reaction and the swelling ratio. The observed increase in the initial rate constant of degrafting with increasing swelling ratio for the PSPMA brushes in aqueous LiCl solution is a first demonstration of this correlation in purely aqueous media, and provides further support for the hypothesis that degrafting of polymer brushes is a mechanochemically catalyzed process driven by a swelling-induced tension at the polymer–substrate interface. For ester hydrolysis in organic media the addition of salt (typically 5–30 equiv with respect to ester) has been reported to affect the rate of hydrolysis.^26,27^ It is important to recall that the experiments conducted here (that were carried out in aqueous media) were performed with 8 × 10 mm^2^ sized planar substrates. The number of hydrolyzable bonds that is present in the monolayer at the brush - substrate interface on these substrates is infinitely small as compared to the number of moles of salt for all concentrations of salt used, so that we assume that for all conditions there is a very large excess of salt as compared to hydrolyzable bonds, and the main effect of varying salt concentrations are the observed changes in the swelling ratios of the polymer brushes.

## Conclusions

This study has explored the salt-dependence of the swelling properties of PSPMA brushes prepared via surface-initiated atom transfer radical polymerization to correlate the swelling ratio of these surface grafted polymers with their degrafting behavior. Analysis of the swollen film thickness of PSPMA brushes immersed in aqueous media containing 5, 50, or 500 mM LiCl or NaCl confirmed the sensitivity of the swelling properties of these brushes toward ionic strength. Variation of the nature of the salt and the salt concentration allowed to conduct degrafting experiments on PSPMA brushes at different swelling ratios. These experiments revealed that in aqueous LiCl solutions, the initial rate constant that describes the degrafting process is correlated with the swelling ratio of the PSPMA brush. This observation represents a first example of the correlation between these two parameters for hydrophilic polymer brushes in aqueous media, and supports the idea that degrafting is a mechanochemically catalyzed process driven by a swelling-induced tension at the polymer–substrate interface. These insights not only add to the fundamental understanding of the degrafting of polymer brushes, but are also relevant from a technological perspective, since polymer brushes are often used in a solvated state, and degrafting thus is related to the robustness and longevity of polymer brush coatings.

## Data Availability

The data underlying this study are available in the Zenodo repository at https://doi.org/10.5281/zenodo.13853762.

## Supporting Information Available

Characterization data of synthesized compounds, raw ellipsometry data, details on the modeling of the ellipsometry data, and results of degrafting experiments (PDF)
