# Supplementary material for: Swelling and Degrafting of Poly(3-sulfopropyl methacrylate) Brushes
Source: Langmuir. 2024 Sep 30;40(41):21656–62. doi: 10.1021/acs.langmuir.4c02714 (PMC11483762; doi:10.1021/acs.langmuir.4c02714)
Supplement: Supplementary file 1 — la4c02714_si_001.pdf [file la4c02714_si_001.pdf]

Supporting Information

Swelling and Degrafting of Poly(3-Sulfopropyl Methacrylate) Brushes

*Sabrina Sant,[1,2] Kuljeet Kaur,[1,2] and Harm-Anton Klok[1,2]**

[1] Institut des Matériaux and Institut des Sciences et Ingénierie Chimiques, Laboratoire des Polymères, École Polytechnique Fédérale de Lausanne (EPFL), Station 12, CH-1015 Lausanne, Switzerland.

[2] National Center of Competence in Research Bio-inspired Materials, Chemin des Verdiers 4, CH-1700, Fribourg, Switzerland

sabrina.sant@epfl.ch, kuljeet.kaur@epfl.ch, harm-anton.klok@epfl.ch

Table of Contents:

Number of pages: 11

Number of figures: 9

Number of tables: 1

CORRESPONDING AUTHOR: Email: harm-anton.klok@epfl.ch; Phone: + 41 21 693 4866

Table S1: Initial degrafting rate constants (k_{init}) determined in aqueous LiCl and NaCl solutions of different ionic strengths with the coefficient of determination from the linear regression. The initial dry film thicknesses of PSPMA brushes grafted from **wafers 1** and **wafer 2** were 66.9 ± 1.5 nm and 56.4 ± 1.4 nm.

**Salt**	**Concentration** [mM]	**Wafer**	k_{init} [h^{-1}]	R^2
LiCl	5	Average	0.025 ± 0.006	
		1	0.02 ± 0.002	0.9671
		2	0.029 ± 0.005	0.9182
LiCl	50	Average	0.046 ± 0.023	
		1	0.068 ± 0.009	0.9686
		2	0.024 ± 0.005	0.9031
LiCl	500	1	0.038 ± 0.004	0.9632
NaCl	5	Average	0.033 ± 0.021	
		1	0.054 ± 0.005	0.9771
		2	0.012 ± 0.002	0.8878
NaCl	50	Average	0.022 ± 0.010	
		1	0.031 ± 0.005	0.9194
		2	0.013 ± 0.002	0.9186
NaCl	500	Average	0.037 ± 0.007	
		1	0.041 ± 0.005	0.9757
		2	0.032 ± 0.007	0.8808

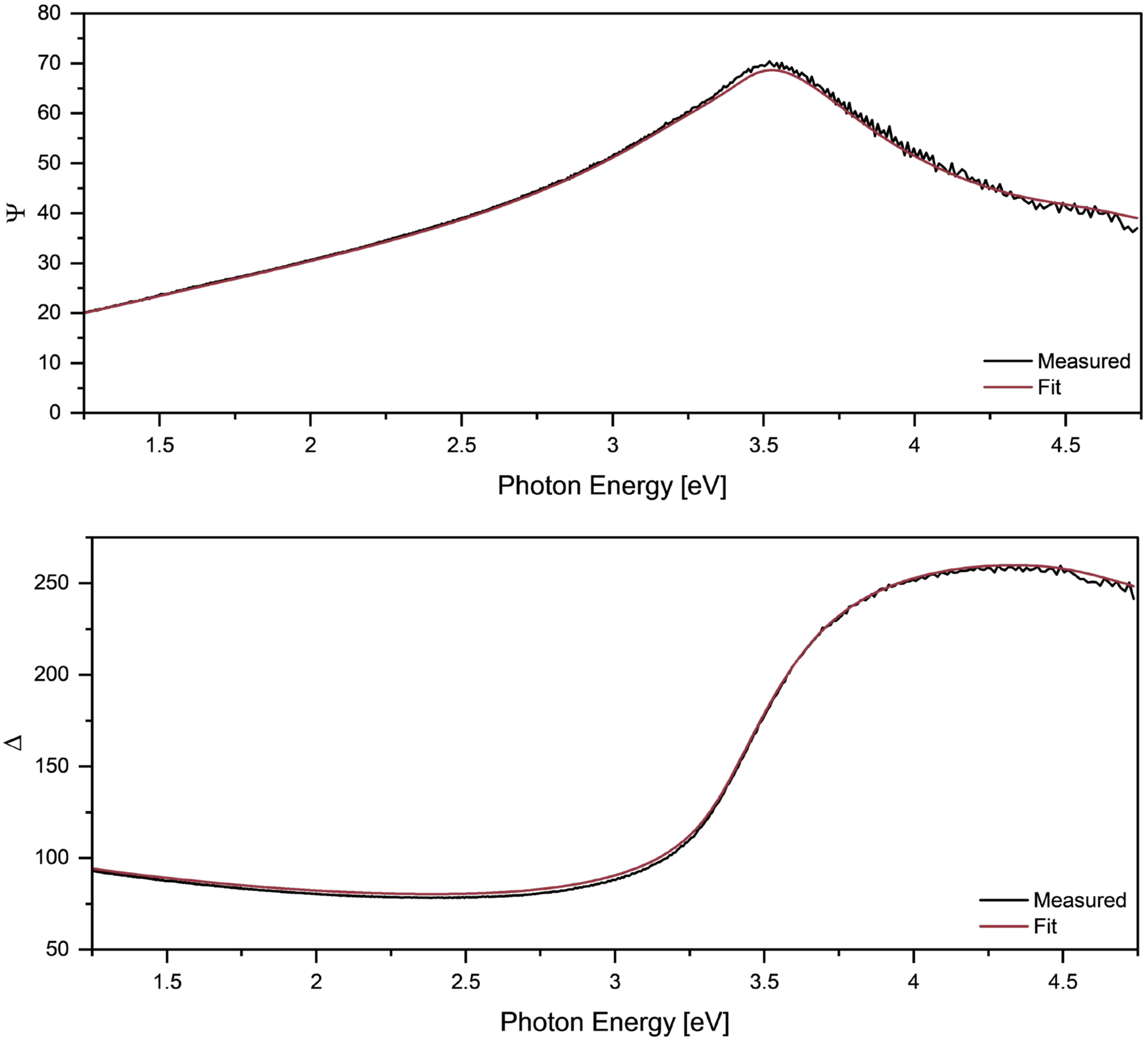

Figure S1: Raw ellipsometric data (black) of a dry PSPMA brush with the corresponding fit (red) for the two parameters Ψ (top) and Δ (bottom).

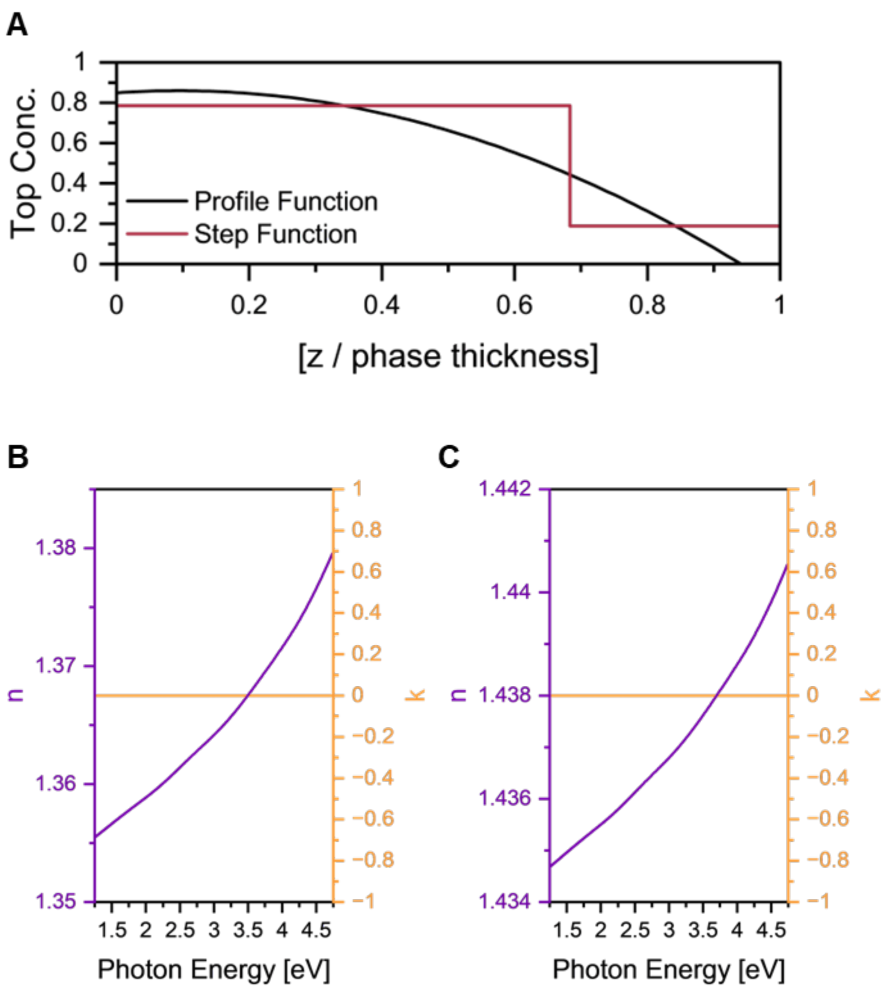

Figure S2: (A) Visualization of the “gradient phase” to describe a swollen polymer brush film in the SEA v1.6.1 software. The transition concentration profile (black) from the top layer to the bottom layer is described by a parabolic function as $c(z) = a(z\text{-}z0)^2 + c0$, where c is the concentration of the top layer and z the gradient phase thickness. Parabola parameters of a = -1.1999, c0 = 0.8598, z0 = 0.0939 were used to model all swollen film thicknesses. The gradient phase was additionally divided into two sublayers. To determine the polymer layer thickness, the thickness and complex refractive index of each sublayer is modeled within the software. The step function (red) shows the relative thickness of each sublayer in the gradient phase of a PSPMA film swollen in 5 mM NaCl. The complex refractive indices of the sublayers result through an effective medium approximation (EMA) of the adjacent layers above and below. (B) The resulting complex refractive index of a PSPMA film swollen in 5 mM NaCl of sublayer 1 and (C) of sublayer 2.

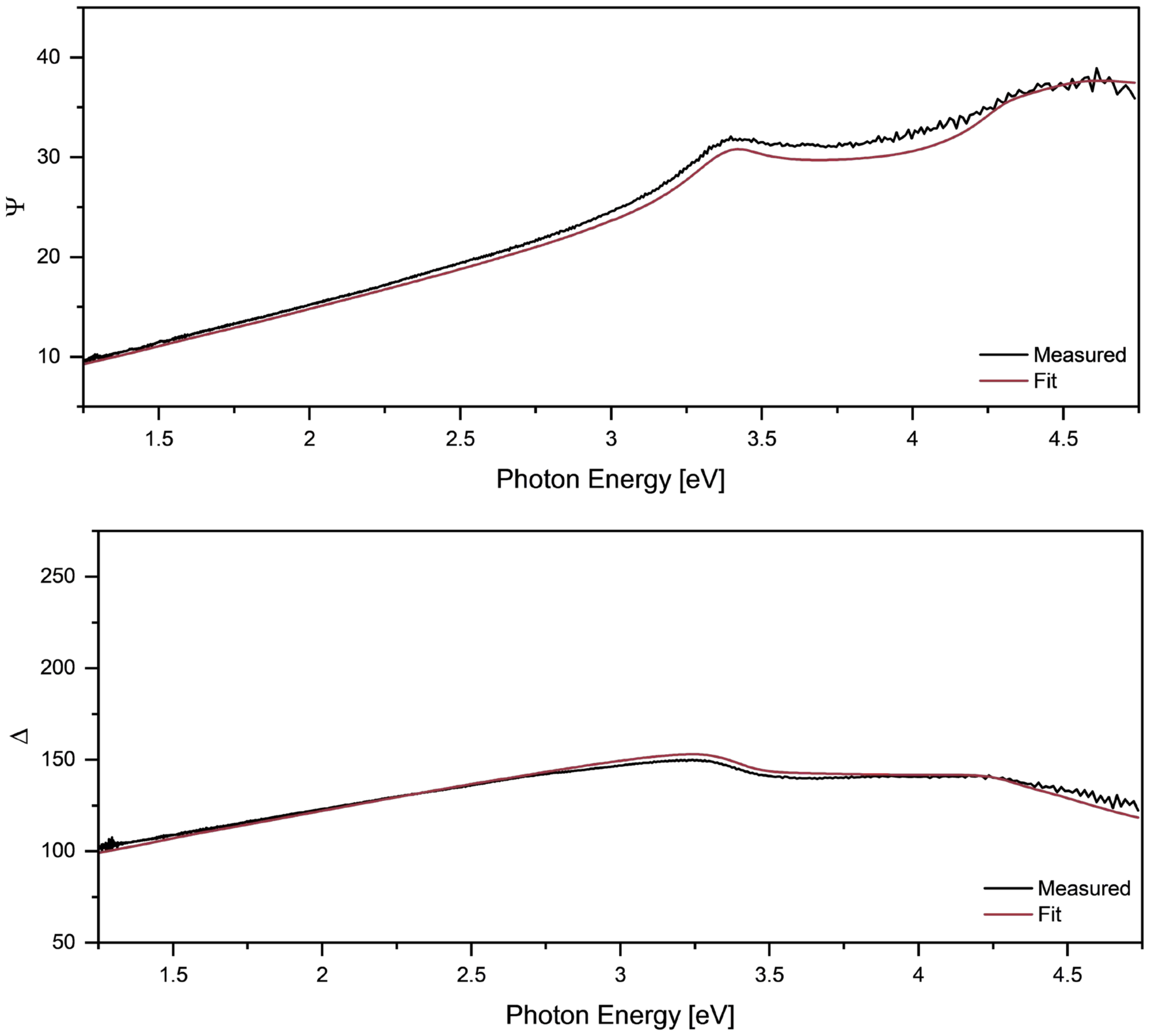

Figure S3: Raw ellipsometric data (black) of a swollen PSPMA brush in 5 mM NaCl with the corresponding fit (red) for the two parameters Ψ (top) and Δ (bottom).

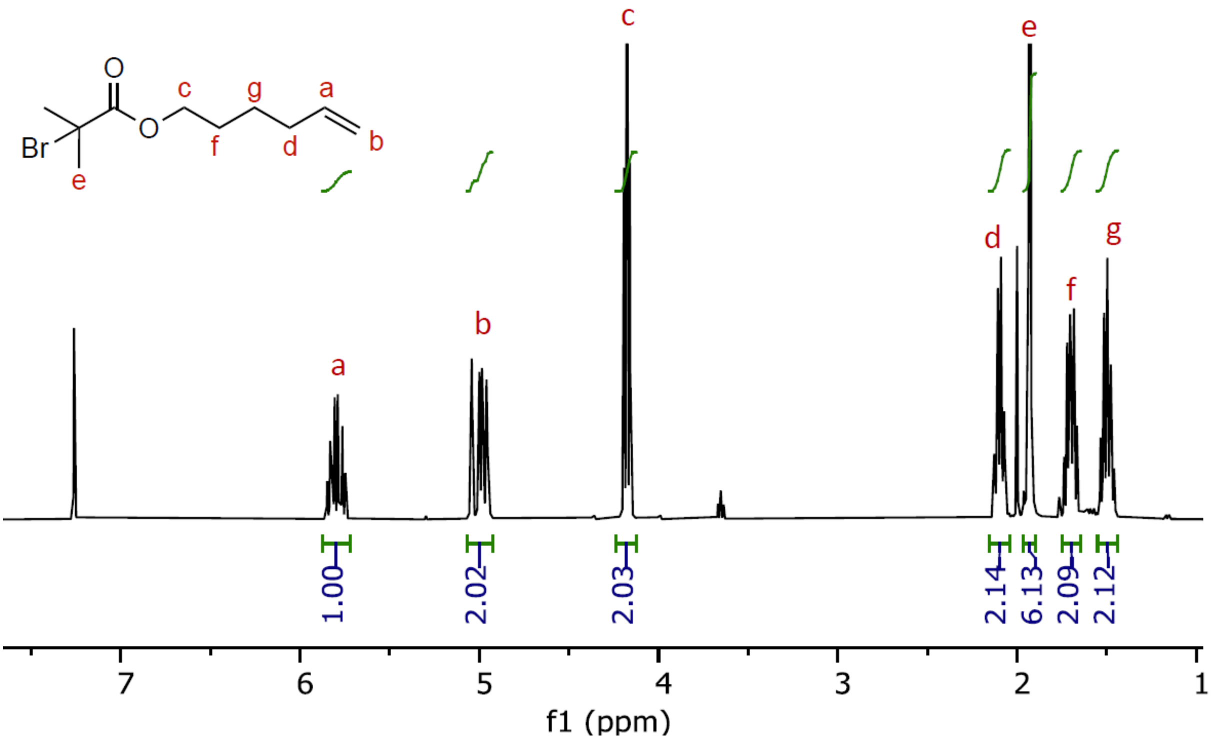

Figure S4: ^{1}H NMR spectrum of 5-hexen-1-yl-2-bromo-2-methylpropionate in $CDCl_3$.

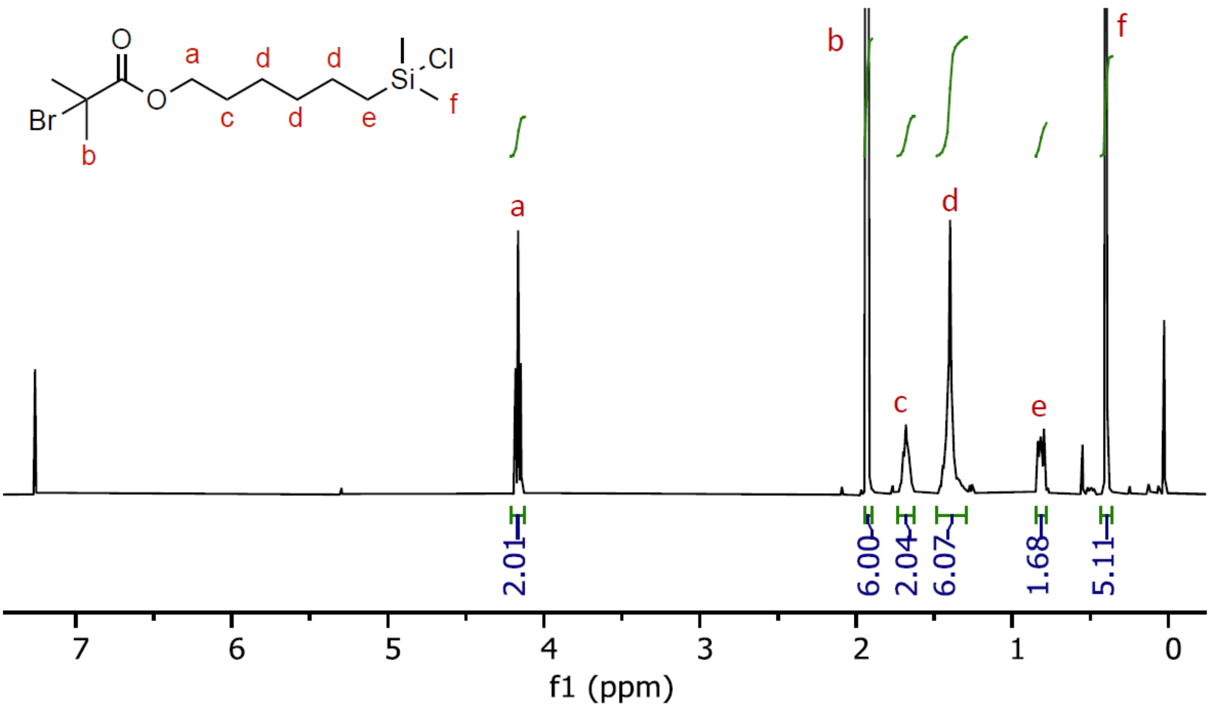

Figure S5: ^{1}H NMR spectrum of 6-(chlorodimethylsilyl)hexyl 2-bromo-2-methylpropanoate in $CDCl_3$.

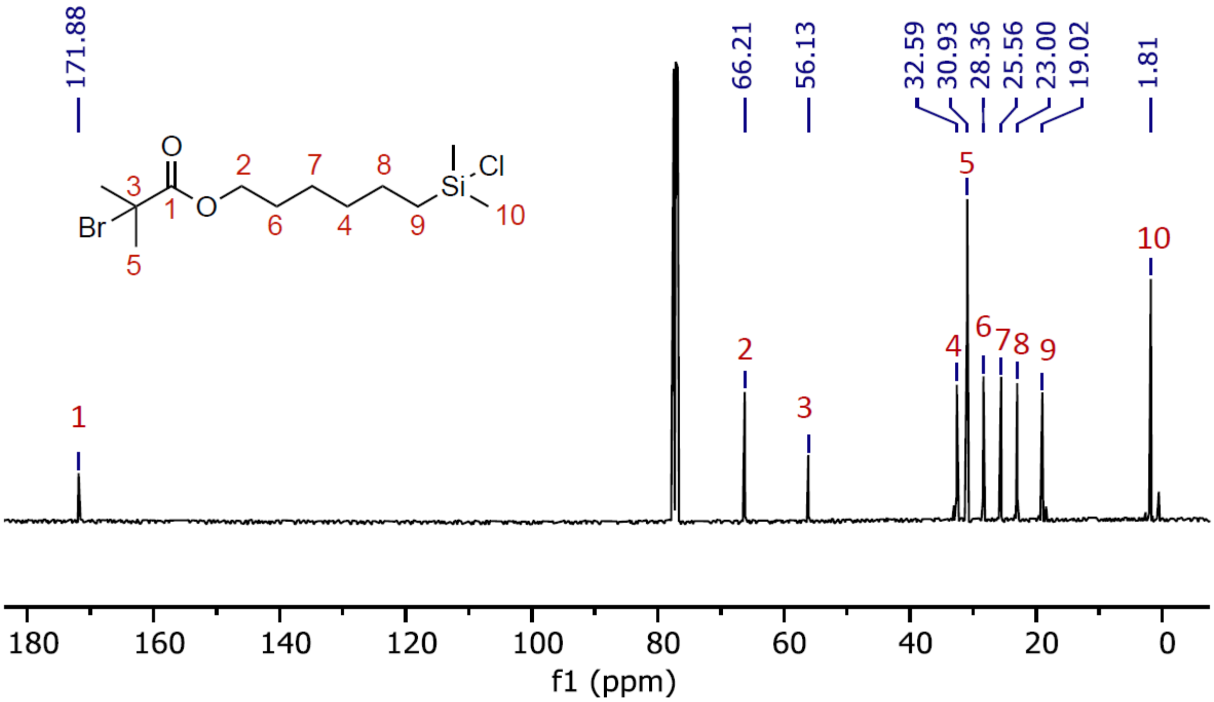

Figure S6: ^{13}C NMR spectrum of 6-(chlorodimethylsilyl)hexyl 2-bromo-2-methylpropanoate in $CDCl_3$.

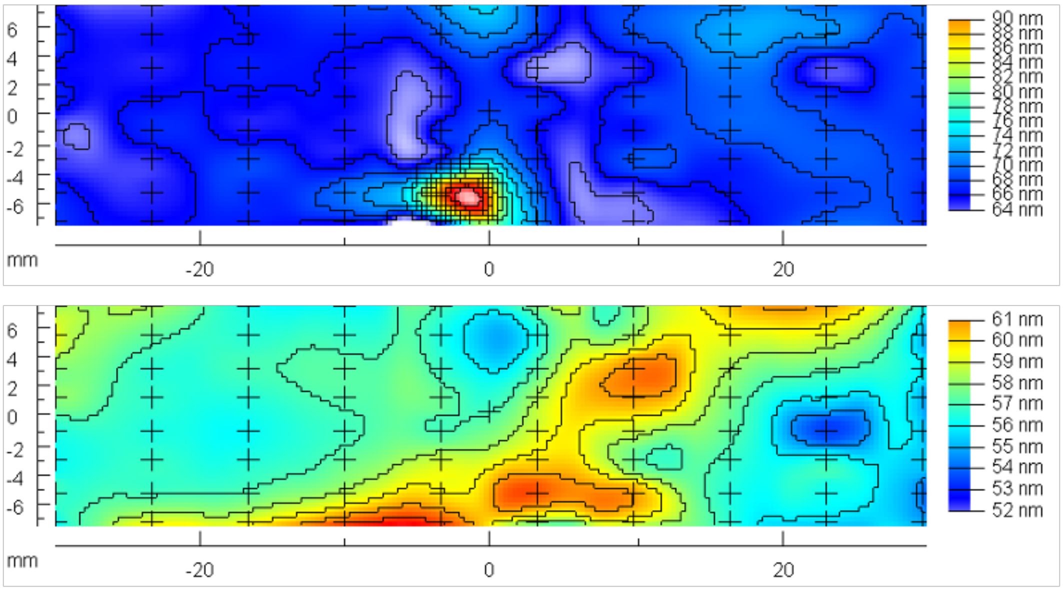

Figure S7: Contour plots of **wafer 1** (top) and **wafer 2** (bottom) covered with PSPMA brushes with dry film thicknesses of 66.9 ± 1.5 nm and 56.4 ± 1.4 nm.

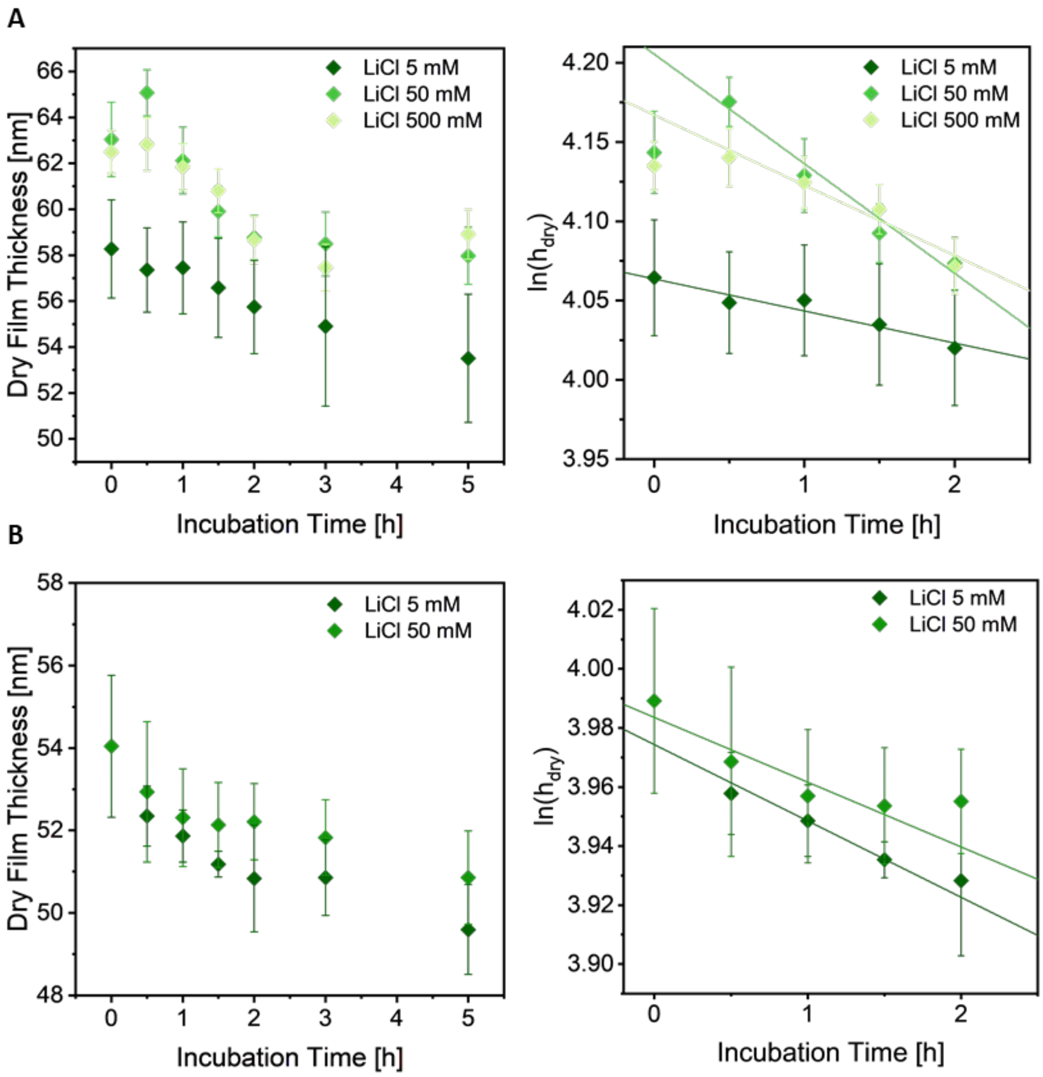

Figure S8: Evolution of dry film thickness and ln(h_{dry}) for PSPMA brushes grafted from **wafer 1** (A) and **wafer 2** (B) as a function of incubation time in LiCl.

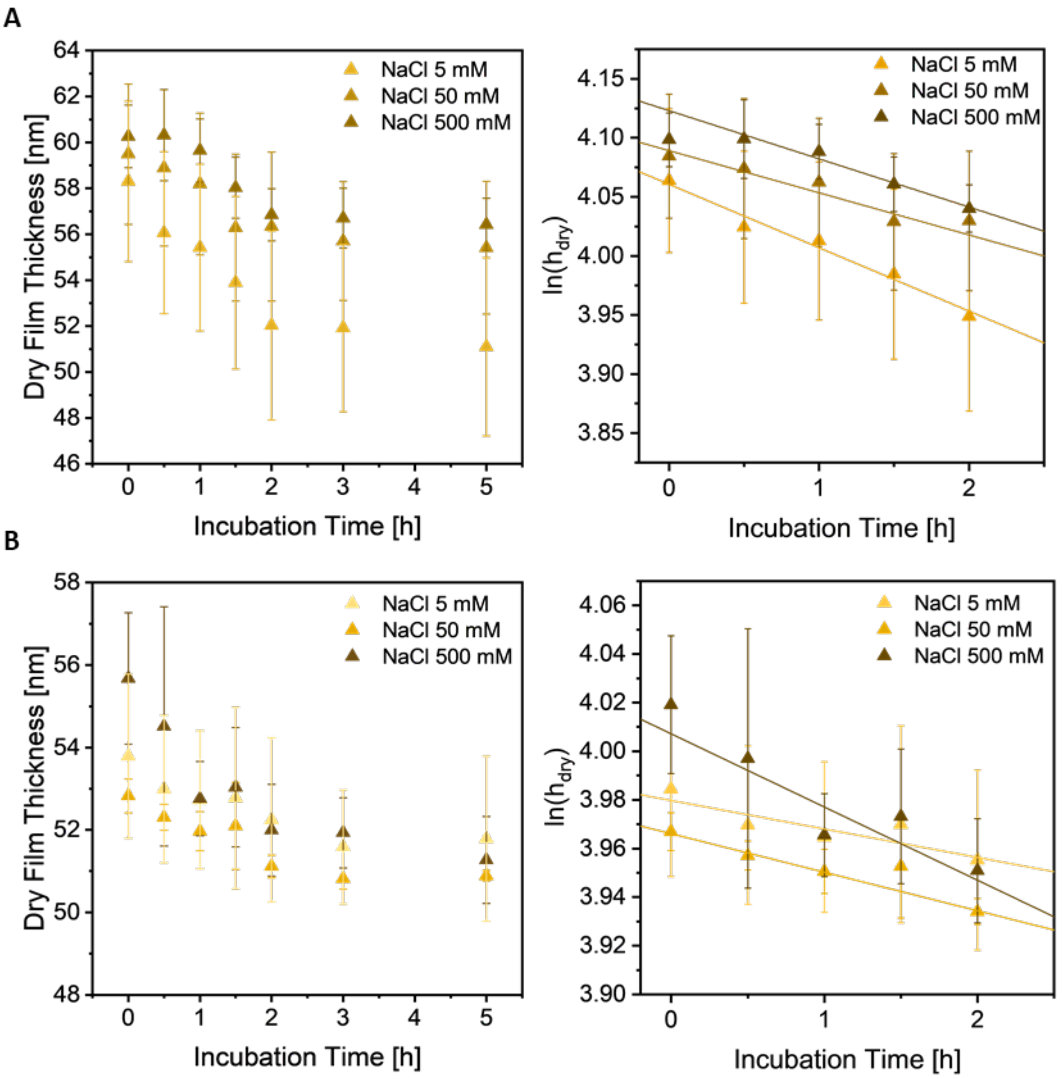

Figure S9: Evolution of dry film thickness and ln(h_{dry}) for PSPMA brushes grafted from **wafer 1** (A) and **wafer 2** (B) as a function of incubation time in NaCl.
